# Pre-application of dentin bonding agent prevents discoloration caused by mineral trioxide aggregate

**DOI:** 10.1186/s12903-020-01151-1

**Published:** 2020-06-03

**Authors:** Yoo-Lim Choi, Young-Eun Jang, Bom Sahn Kim, Jin-Woo Kim, Yemi Kim

**Affiliations:** 1 Department of Conservative Dentistry, College of Medicine, Ewha Womans University, 1071, Anyangcheon-ro, Yangcheon-gu, Seoul, 07985 South Korea; 2 Department of Nuclear Medicine, College of Medicine, Ewha Womans University, Seoul, South Korea; 3 Department of Oral & Maxillofacial Surgery, College of Medicine, Ewha Womans University, Seoul, South Korea

## Abstract

**Background:**

To evaluate tooth discoloration by newly developed calcium silicate-based materials, and to examine the pre-application of dentin bonding agent (DBA) for preventing discoloration caused by mineral trioxide aggregate (MTA).

**Methods:**

The roots of 50 premolars were randomly divided into five groups (*n* = 10) and cavities were prepared from resected root surfaces. MTA was placed in the cavities of teeth belonging to the ProRoot MTA (MTA) and RetroMTA (RMTA) groups. For teeth belonging to the ProRoot + DBA (MTA-B) and RetroMTA + DBA (RMTA-B) groups, DBA was first applied to the cavities prior to the addition of MTA. Teeth in the control group were restored with composite resin only (i.e., without MTA). After 12 weeks, MTA was removed from the MTA and RMTA groups and bleaching agents were applied for 3 additional weeks. Color assessments were recorded at baseline, and 1, 4, and 12 weeks, as well as after bleaching. A one-way ANOVA was performed to assess the differences between the two types of MTAs and color changes following DBA pre-application in each MTA group. A *p*-value of < 0.05 was considered indicative of statistical significance.

**Results:**

Following 12 weeks of MTA treatment, there was a significant difference between the discoloration in the MTA and RMTA groups (*p* < 0.05). However, no significant difference was observed between the RMTA and RMTA-B groups (*p* > 0.05). Following bleaching, the color changes (ΔE values) of the MTA group were not significantly different from those of the MTA-B group (*p* > 0.05). The difference of ΔE between the RMTA group after internal bleaching and the RMTA-B group was also not significant (*p* > 0.05).

**Conclusions:**

RetroMTA caused significantly less discoloration than ProRoot MTA. Pre-application of DBA reduced discoloration caused by ProRoot MTA. MTA discoloration was improved equally well between DBA pre-application and post-bleaching.

## Background

Mineral trioxide aggregate (MTA) is the material of choice for endodontic treatments because it is a biocompatible repair material with high sealing ability [1, 2]. MTA is used for non-surgical and surgical purposes, including root-end filling, perforation repair, direct pulp capping, and repair of teeth with open apices [3, 4]. It is biocompatible, has less leakage than other materials, and reportedly promotes the formation of hard tissue [5–9].

Despite its favorable properties, it has a prolonged setting time and causes discoloration [10, 11]. MTA was first derived from Portland cement and introduced in a gray form (GMTA). However, it caused coronal tooth discoloration, necessitating the development of white MTA (WMTA). Compared to GMTA, WMTA has lower concentrations of aluminum oxide, magnesium oxide, and ferrous oxide, which are responsible for tooth discoloration [12]. Coronal discoloration by WMTA has been detected in in vitro and ex vivo studies [13, 14]. Besides metal oxides, the causes of MTA discoloration are the contamination of MTA by blood [10, 15], the interaction between MTA and sodium hypochlorite (NaOCl) [16, 17], and the effects of light and oxygen [15, 18].

To improve the properties of ProRoot MTA (Dentsply Endodontics, Tulsa, OK), multiple calcium-silicate-based materials have been developed, including Biodentine (Septodont, Saint Maur des Faussés, France), BioAggregate (Innovative Bioceramix, Vancouver, BC, Canada), Endocem MTA (Maruchi, Wonju, South Korea), MTA Angelus (Angelus, Londrina, PR, Brazil), and RetroMTA (BioMTA, Seoul, South Korea). RetroMTA is a hydraulic bioceramic material used in vital pulp therapy but is not derived from Portland cement. RetroMTA has an initial setting time of 150 s and does not cause discoloration, even when combined with blood [19]. Thus, RetroMTA may be suitable for esthetic repair purposes.

ProRoot MTA contains 44.2% calcium oxalate, 21.2% silicon dioxide, 16.1% bismuth oxide, 1.9% aluminum oxide, 1.4% magnesium oxide, 0.6% sulfur trioxide, and 0.4% ferrous oxide. Conversely, RetroMTA contains 60–80% calcium carbonate, 5–15% silicon dioxide, 5–10% aluminum oxide, and 20–30% calcium zirconia complex, which acts as a radiopacifier (RetoMTA).

The development of dentin bonding systems and adhesive resins is among the most important areas of study in operative dentistry. One recent study showed that applying two layers of dentin bonding agent (DBA) prior to WMTA or GMTA could prevent tooth discoloration [20]. Although the samples showed varying degrees of discoloration, prior application of DBA reduced the discoloration caused by MTA. Also, the sealing ability of DBA prevented penetration of MTA into dentinal tubules. The purpose of this study was to evaluate the discoloration of ProRoot MTA and RetroMTA, and to evaluate the effects of using a dentin adhesive prior to the addition of MTA. We also compared the effects of the DBA pre-application and bleaching on MTA discoloration.

## Methods

### Sample preparation

This study was approved by the Institution Ethics Committee. Fifty human premolars were extracted in accordance with the orthodontic treatment plan and stored in a physiologic saline solution until further use. The teeth were clinically and radiographically examined before and after the extraction procedures. The inclusion criteria for the samples were as follows: free of caries, restoration, cracks, and calcification. Tooth samples that met any of the following criteria were excluded: teeth extracted due to periodontal diseases; teeth with open apices; teeth with root canal fillings, posts, crown restorations, or any metallic restorations; cracked teeth; teeth with any signs of internal or external resorption; and failure to provide informed consent.

Using a high-speed diamond bur with water coolant, roots were resected 3 mm below the cemento-enamel junction. Following this, an access cavity was prepared from the resected root surface in the coronal direction using an Endo Z bur (Dentsply Maillefer, Ballaigues, Switzerland). After preparation of an access cavity, the canals were irrigated by using a 2.5% sodium hypochlorite solution for cavity cleaning.

Teeth were randomly assigned to the following groups:

1. ProRoot MTA (MTA group)
2. RetroMTA (RMTA group)
3. ProRoot MTA with prior application of DBA (MTA-B group)
4. RetroMTA with prior application of DBA (RMTA-B group)
5. Control group

For the ProRoot MTA (MTA) and RetroMTA (RMTA) groups, MTA plugs were inserted into the cavities, up to the cemento-enamel junction. For the ProRoot MTA with prior application of DBA (MTA-B) and RetroMTA with prior application of DBA (RMTA-B) groups, two layers of dentin adhesive (AdheSE; Ivoclar Vivadent, Schaan, Liechtenstein) were applied to the cavity according to the manufacturer’s instructions; AdheSE primer was applied for 30 s, and the excess primer was dispersed with a strong stream of air. Following primer, the AdheSE bonding agent was applied, and the excess was scattered with a soft flow of air. Light cured was done for 40 s. Following curing, MTA plugs were inserted into the cavities up to the cemento-enamel junction.

Following cleaning of the cavities, wet cotton pellets were placed over the MTA plugs and cavities were sealed with temporary filling materials (Caviton; GC Corp, Tokyo, Japan). Teeth were then stored in physiological saline for 1 day. The temporary filling materials were then removed, and MTA plugs were inspected to assess curing. Teeth were then restored with composite resin (Tetric ceram A3; Ivoclar Vivadent, Schaan, Liechtenstein). Teeth without MTA were restored with only composite resin (control teeth). All specimens were immersed in saline at room temperature and replenished every week.

### Measurement of tooth discoloration

Tooth color changes were recorded using a spectrophotometer (SpectroShade™ Micro; MHT Medical High Technologies, Verona, Italy) at the outset of experimentation, and after 1, 4, and 12 weeks. Before spectrophotometry, an observer calibrated the spectrophotometer according to the manufacturer’s recommendations. The same operator performed measurements under continuous laboratory illumination by positioning the spectrophotometer optimally at the green line and obtaining three measurements. The average of the three measurements was used for subsequent analyses. The data were expressed using the CIE L*a*b* system, where L* represents the shade ranging from black (0) to white (100), and a* and b* correspond to chromaticity from red (+80a*) to green (−80a*) and yellow (+80b*) to blue (−80b*), respectively. Color differences between the baseline and each measurement were expressed as ΔE, based on the following:

$$ \Delta  \mathrm{E}={\left[{\left({L}_2-{L}_1\right)}^2+{\left({a}_2-{a}_1\right)}^2+{\left({b}_2-{b}_1\right)}^2\right]}^{1/2} $$

Delta values ≥3.3 were considered clinically unacceptable [21].

### Internal bleaching of discolored teeth

The MTA and RMTA groups were used to test the effects of bleaching on MTA discoloration. Following 12 weeks of treatment, the composite resin restorations and MTA were removed using a low-speed #1 carbide bur and a microscope (Zeiss OPMI pico; Carl Zeiss, Göttingen, Germany). A sodium perborate and distilled water solution (2:1 ratio) was used as the bleaching agent [22], which was condensed in the access cavities and sealed with temporary filling materials. Bleach was applied for 3 weeks with replacement each week. Chromatic changes were measured following the removal of MTA and bleaching for 1, 2, and 3 weeks.

### Statistical analysis

Statistical analyses were performed using SPSS software (SPSS statistics 21.0; SPSS Inc., Chicago, IL). A one-way ANOVA was used to assess the differences between the MTAs at each time point and color changes following DBA pre-application in each MTA group. Post hoc Tukey’s tests were used for pairwise comparisons. A *p*-value of < 0.05 was considered statistically significant.

## Results

### Measurement of tooth discoloration

**Fig. 1:**
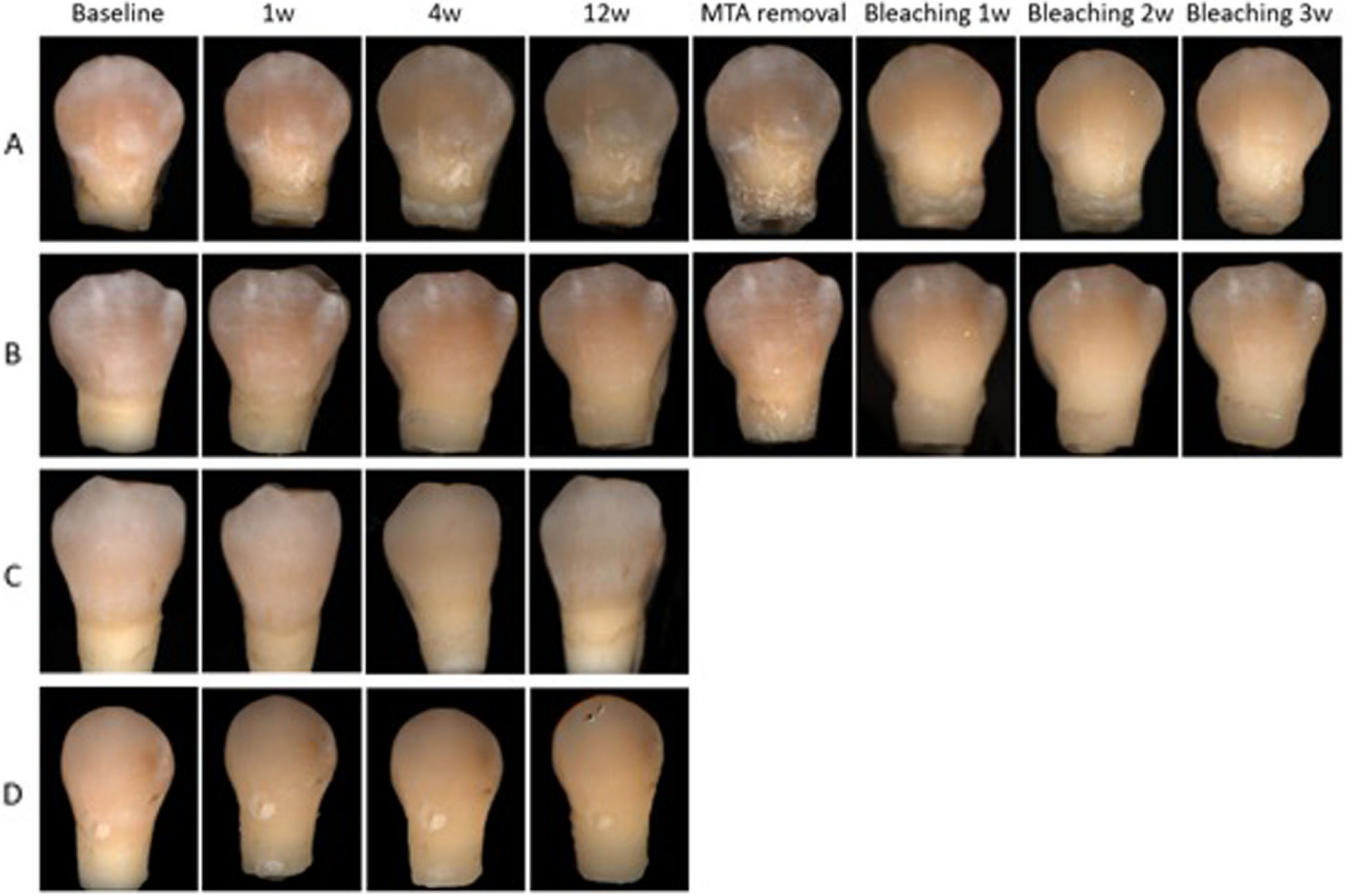
Photographs of color changes in teeth over the study period. **a** ProRoot MTA group; **b** RetroMTA group; **c** DBA + ProRoot MTA group; **d** DBA + RetroMTA group. DBA, dentin bonding agent; w, week

Figure 1 shows the color changes for all groups over the 12-week experimentation period. Tooth samples from the MTA group had the most pronounced color changes, while discoloration in the RMTA group was less pronounced. Teeth belonging to the MTA-B group had less discoloration than teeth in groups lacking the bonding agent. The RMTA-B group had the least discoloration of all groups. The mean and standard deviation values for all groups and the controls are shown in Table 1. The L* and a* values decreased in all groups following the 12-week treatment period, which was most pronounced in the MTA group (ΔL* = 10.44, Δa* = 2.49). Additionally, the average b* values decreased in the MTA group, whereas they increased in all other groups.

**Table 1:** The CIE L*, a*, and b* chromatic parameters, and ΔE values for different groups following MTA treatment

CIE L^*^ parameter		Baseline	1 week	4 weeks	12 weeks
Groups	n	L_0_	L_1_	L_2_	L_3_
Control	10	78.45 (1.62)	75.48 (2.16)	76.09 (2.02)	74.53 (1.91)
ProRoot	10	80.15 (1.78)	75.01 (1.72)	73.27 (1.77)	69.71 (1.65)
Retro	10	80.69 (1.45)	78.96 (1.94)	78.28 (1.30)	77.13 (1.65)
DBA + ProRoot	10	78.65 (1.27)	75.29 (1.69)	73.44 (1.36)	72.70 (1.66)
DBA + Retro	10	78.08 (1.15)	75.65 (1.61)	74.51 (1.03)	74.91 (1.11)
CIE a^*^parameter		Baseline	1 week	4 weeks	12 weeks
Groups	n	a_0_	a_1_	a_2_	a_3_
Control	10	3.43 (0.91)	2.94 (0.87)	3.11 (0.82)	2.27 (0.77)
ProRoot	10	3.52 (0.85)	2.41 (0.90)	1.40 (0.71)	1.03 (0.69)
Retro	10	2.73 (0.65)	2.73 (0.85)	2.09 (0.70)	2.01 (1.09)
DBA + ProRoot	10	2.78 (0.59)	2.25 (0.61)	1.62 (0.51)	1.42 (0.69)
DBA + Retro	10	2.95 (0.66)	2.33 (0.79)	2.29 (0.92)	2.62 (0.90)
CIE b* parameter		Baseline	1 week	4 weeks	12 weeks
Groups	n	b_0_	b_1_	b_2_	b_3_
Control	10	19.57 (1.73)	20.81 (1.29)	19.56 (1.47)	22.55 (1.23)
ProRoot	10	18.05 (1.75)	17.6 (2.78)	17.23 (2.31)	16.91 (2.52)
Retro	10	17.54 (2.93)	19.49 (3.50)	19.82 (4.55)	21.43 (3.46)
DBA + ProRoot	10	16.83 (2.08)	17.06 (2.11)	19.83 (2.36)	18.7 (2.89)
DBA + Retro	10	17.83 (1.83)	20.89 (2.08)	22.89 (1.93)	22.73 (2.22)
**ΔE value**			1 week	4 weeks	12 weeks
Groups	n		ΔE_1_	ΔE_2_	ΔE_3_
Control	10		7.65 (5.28)^a^	6.66 (5.47)^a^	16.41 (8.07)^a^
ProRoot	10		16.05 (10.05)^ab^	27.98 (10.63)^b^	61.45 (24.08)^b^
Retro	10		10.97 (9.20)^a^	21.87 (13.13)^b^	19.30 (12.02)^a^
DBA + ProRoot	10		7.06 (4.86)^a^	20.55 (7.30)^b^	23.88 (10.65)^a^
DBA + Retro	10		5.15 (5.03)^ac^	8.40 (8.19)^a^	16.69 (8.97)^a^

Data are expressed as the mean (SD)

Same letters indicate that the values are statisticallly similar for each column (*p* < 0.05)

*DBA* dentin bonding agent

**Fig. 2:**
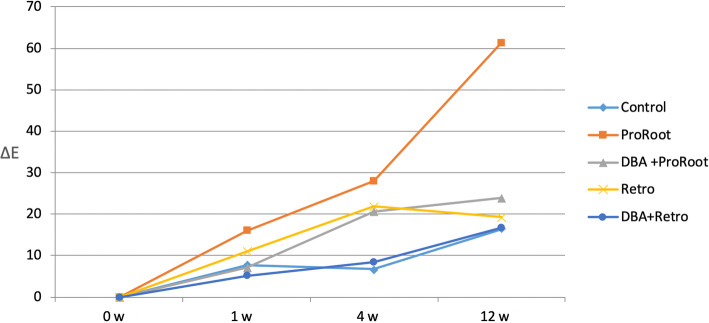
Changes in the ΔE values of the experimental and control groups during the 12-week MTA treatment period

The average ΔE values for each group are shown in Table 1 and Fig. 2. Notably, the ΔE increased the most among teeth in the MTA group, and the difference in ΔE between the MTA and MTA-B groups was statistically significant (*p* < 0.05). No difference in the ΔE between the RMTA and RMTA-B groups was observed (*p* > 0.05). After 4 weeks, there were significant differences in ΔE values between the MTA group and the MTA-B group and between the RMTA group and the RMTA-B group (*p* < 0.05). At the end of the examination period, the difference in ΔE values between the MTA group and MTA-B group was significant, but that between the RMTA group and the RMTA-B group was not (*p* > 0.05).

### Internal bleaching of discolored teeth

**Fig. 3:**
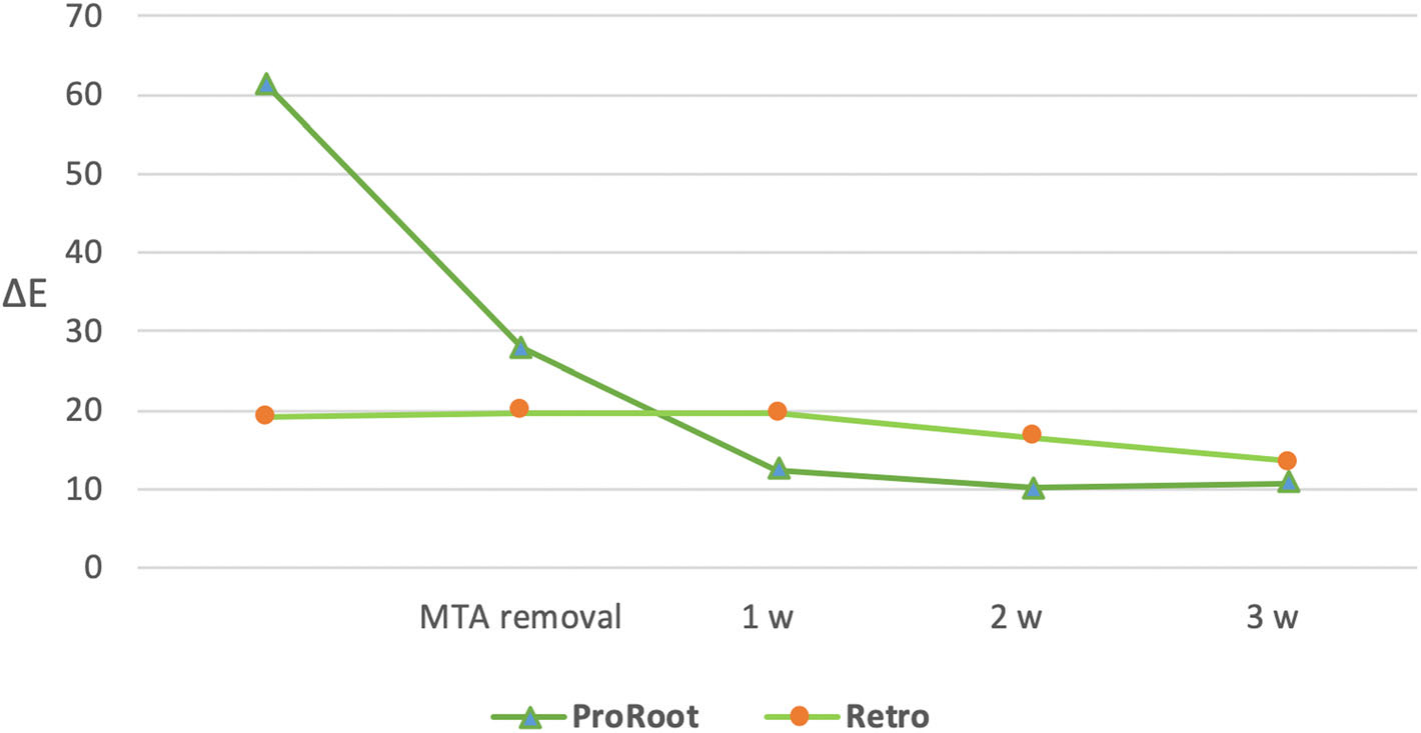
Changes in ΔE values due to bleaching following the removal of MTA

Following 12 weeks of treatment in the MTA and RMTA groups, MTA was removed and internal bleaching was performed for 3 weeks. Following the removal of MTA from the MTA group, but prior to bleaching, a significant difference in ΔE was observed. After 3 weeks of bleaching, ΔE values decreased in both groups and there was no significant difference between the two groups after the bleaching procedure (Table 2, Fig. 3).

**Table 2:** CIE L*, a*, and b* parameters, and ΔE following bleaching

CIE L^*^ parameter		MTA removal	1 week	2 weeks	3 weeks
Groups	n	L_0_	L_1_	L_2_	L_3_
ProRoot	10	73.3 (2.30)^a^	78.18 (1.50)^a^	79.27 (0.94)^a^	81.00 (1.29)^a^
Retro	10	76.7 (0.97)^b^	78.71 (1.46)^a^	79.39 (0.94)^a^	81.55 (1.05)^a^
CIE a^*^parameter		MTA removal	1 week	2 weeks	3 weeks
Groups	n	a_0_	a_1_	a_2_	a_3_
ProRoot	10	1.54 (0.63)^a^	0.72 (0.59)^a^	−0.08 (0.63)^a^	−0.17 (0.58)^a^
Retro	10	2.68 (0.73)^b^	1.02 (0.66)^a^	−0.04 (0.67)^a^	−0.23 (0.55)^a^
CIE b^*^parameter		MTA removal	1 week	2 weeks	3 weeks
Groups	n	b_0_	b_1_	b_2_	b_3_
ProRoot	10	17.65 (2.61)^a^	20.81 (1.75)^a^	19.13 (1.48)^a^	18.03 (1.63)^a^
Retro	10	20.47 (2.33)^b^	21.80 (1.50)^a^	20.44 (1.79)^a^	19.49 (1.46)^a^
**ΔE**		MTA removal	1 week	2 weeks	3 weeks
Groups	n	ΔE_0_	ΔE_1_	ΔE_2_	ΔE_3_
ProRoot	10	27.82 (13.44)^a^	12.38 (8.49)^a^	10.22 (4.79)^a^	10.73 (4.29)^a^
Retro	10	19.77 (12.33)^b^	19.58 (20.16)^a^	16.51 (15.77)^a^	13.53 (15.78)^a^

Same letters indicate that the values are statistiicallly similar for each column (*p* < 0.05)

Data are expressed as the mean (SD)

**Fig. 4:**
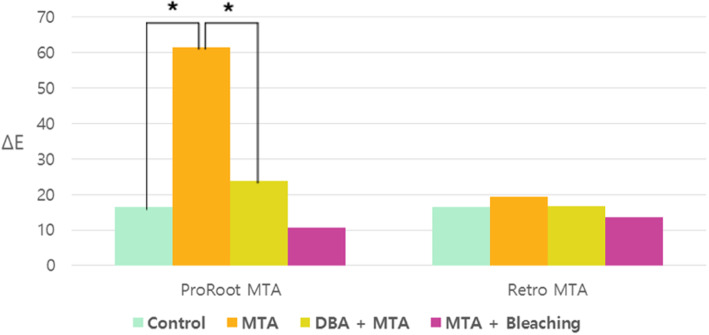
Comparison between the pre-application of dentin-bonding agent and bleaching on tooth discoloration. *p < 0.05

Significantly less discoloration was observed in the MTA group, which received DBA pre-application. Moreover, when bleaching was performed in teeth that did not receive DBA, the ΔE value was smaller than that of the MTA-B group (not significant) (Fig. 4).

No significant difference in color change was observed between the RMTA group and control teeth. Additionally, no significant difference was observed between teeth receiving DBA pre-application and those that did not receive DBA, nor was any difference observed between teeth receiving DBA or bleaching (Fig. 4).

## Discussion

MTA shows improved outcomes in endodontics as a result of its excellent biocompatibility [4, 23], good sealing ability [24], low leakage [25], and promotion of hard tissue formation [26]. However, the tooth discoloration induced by MTA is one of its drawbacks. For the prevention and treatment of discoloration, previous studies have suggested placing a double layer of DBA over the dentine in an access cavity before applying MTA [14] and internal bleaching after removing MTA [11]. To the best of our knowledge, only one previous study [14] has investigated the application of adhesive materials prior to use of MTA inside the pulp chamber. Moreover, no study has compared the degree of tooth discoloration following placement of ProRoot MTA and other recently developed tricalcium silicate cements or investigated the effect of prior application of DBA on these materials. Therefore, our findings provide information useful for preventing the discoloration induced by MTA.

Following the 12-week treatment period, tooth shade changed in nearly all teeth, including the control teeth. Teeth from the MTA group displayed the most extreme color change to gray, while the MTA-B teeth exhibited only marginal changes. Additionally, the degree of discoloration was significantly less in the RMTA group than in teeth in the MTA group. Analyses of the CIE L*a*b* parameters revealed that tooth lightness decreased in all groups after 12 weeks, with the greatest reduction observed in the MTA group (ΔL* = 10.44). Notably, the large reduction in ΔL for the MTA group was due to its substantial reduction in gray discoloration. The a* and b* values were indicative of redness and yellowness, respectively. The a* was reduced in all groups, with the greatest reduction in the MTA group (Δa* = 2.49). Moreover, b* increased in all groups except MTA, in which it decreased (Δb* = 1.14). These data were consistent with the observation of gray and dark blue discolorations in the MTA group.

One previous study evaluating the discoloration of GMTA and WMTA revealed that discoloration occurred within the MTA directly rather than in the dentin. In that study, crown shade also improved when MTA was removed [23, 25]. However, discolored byproducts remained in the dentin following the removal of MTA, which may have contributed to coronal discoloration. Another study revealed that the addition of two layers of DBA prior to MTA treatment prevented discoloration [20]. In cases when discoloration occurred, removal of MTA and bleaching required caution and additional operational procedures. Thus, we hypothesized that the pre-application of DBA could achieve the same effect as the removal of MTA and bleaching. Therefore, we compared the pre-application of DBA to post-bleaching in ProRoot MTA-treated teeth.

In the MTA group, the ΔE calculated on the basis of the L*, a*, and b* parameters continuously increased. The magnitude of the increase in ΔE was significantly smaller in the MTA-B group than the MTA group. This result was consistent with the findings of a previous study that suggested that DBA may prevent discoloration by ProRoot MTA [20]. DBA may seal the dentinal tubules and prevent MTA penetration. In the RMTA group, ΔE increased following the 12-week treatment period, but was not significantly different from that in the control group. Additionally, pre-application of DBA did not prevent tooth discoloration. The lower discoloration by RetroMTA than by ProRoot MTA was due to their differences in composition. RetroMTA does not contain metal oxides, which cause the discoloration by ProRoot MTA. RetroMTA also includes calcium zirconia complex as a radiopacifier instead of bismuth oxide. Thus, it could be suggested that, because RetroMTA-treated teeth had low discoloration, the DBA pre-application was less effective.

The removal of composite materials in the MTA and RMTA groups confirmed that the MTA itself darkened over time. This is particularly true for ProRoot MTA. Following the removal of MTA, dark discoloration spreading to the dentin was observed at the MTA-dentin interface. This finding is consistent with that of a previous study where staining of the complete dentin wall of the pulp chamber was observed in both WMTA and GMTA with staining penetrating into the dentinal tubule [23]. Recent in vitro studies showed that by-products of MTA hydration accumulate on the surface of the material or on the MTA-dentin interface and intratubular dentin [27, 28]. Another study suggested that calcium released from the MTA reacted with phosphate ions in the tissue fluid, causing precipitation of the carbonated apatite [29]. It was hypothesized that MTA constituents bound phosphate ions or plasma proteins in the dentinal fluid and that the byproducts were oxidized and transformed into pigmented byproducts [25]. Pre-application of DBA protects dentin from MTA powders, which induce contamination or discoloration. Additionally, this process prevents the MTA from reacting with the ions in the tissue fluid and prohibits byproducts from penetrating the dentinal tubules.

The MTA and RMTA groups were used to test the effects of bleaching on MTA discoloration. In the MTA group, the removal of the discolored MTA prior to the application of the bleaching agent resulted in the reversal of discoloration. Upon bleaching of the remaining discolored dentin in teeth from the MTA group, the ΔE values were smaller than those of the MTA-B group. However, this difference was not significant. This comparison is also limited since the results depended on the timing and duration of the bleaching procedure in the MTA group. Thus, DBA pre-application is recommended for esthetic purposes; however, bleaching can also be used if DBA pre-application is not performed.

One recent study revealed that bleaching of MTA may destroy the MTA surface due to the acidic pH [30]. Therefore, pre-application of DBA may be a superior treatment option prior to treatment with ProRoot MTA. Conversely, the use of DBA may be limited since the cytotoxicity of the DBA monomer may affect pulp tissue [31]. DBA may also interfere with the calcium releasing capacity of MTA or its sealing ability. Further studies are required to evaluate the potential limitations, and although DBA may influence the pulp and MTA, detailed studies are lacking. Therefore, to prevent complications associated with DBA, methods should be devised to protect pulp before the application of DBA.

## Conclusions

RetroMTA caused significantly less discoloration than ProRoot MTA. The addition of DBA prior to treatment with ProRoot MTA reduced discoloration; however, no difference was observed when DBA was pre-applied to teeth receiving RetroMTA. Moreover, no significant difference in MTA discoloration was observed between the pre-application of DBA and the post-bleaching procedure. Prior use of adhesives to occlude dentinal tubules could reduce the discoloration induced by MTA.

## Data Availability

The datasets can be accessed on 10.5281/zenodo.3842663

## Abbreviations

MTA: Mineral trioxide aggregate
DBA: Dentin bonding agent
MTA group: ProRoot MTA group
RMTA group: RetroMTA group
MTA-B: ProRoot MTA with prior application of DBA
RMTA-B: RetroMTA with prior application of DBA
